# Oxidative Stress in Chronic Liver Disease and Portal Hypertension: Potential of DHA as Nutraceutical

**DOI:** 10.3390/nu12092627

**Published:** 2020-08-28

**Authors:** Zoe Boyer-Diaz, Paloma Morata, Peio Aristu-Zabalza, Albert Gibert-Ramos, Jaime Bosch, Jordi Gracia-Sancho

**Affiliations:** 1 Barcelona Liver Bioservices, 08034 Barcelona, Spain; zoe@liver.barcelona (Z.B.-D.); peio@liver.barcelona (P.A.-Z.); 2 BrudyTechnology, 08006 Barcelona, Spain; pmorata@brudylab.com; 3 Liver Vascular Biology Research Group, IDIBAPS, 08036 Barcelona, Spain; albertgibert55@hotmail.com (A.G.-R.); jbosch@clinic.cat (J.B.); 4 CIBEREHD, 28029 Madrid, Spain; 5 Hepatology, Department of Biomedical Research, University of Bern, 3012 Bern, Switzerland

**Keywords:** TG-DHA, omega-3, oxidative stress, cirrhosis, liver fibrosis, inflammation

## Abstract

Chronic liver disease constitutes a growing public health issue worldwide, with no safe and effective enough treatment clinical scenarios. The present review provides an overview of the current knowledge regarding advanced chronic liver disease (ACLD), focusing on the major contributors of its pathophysiology: inflammation, oxidative stress, fibrosis and portal hypertension. We present the benefits of supplementation with docosahexaenoic acid triglycerides (TG-DHA) in other health areas as demonstrated experimentally, and explore its potential as a novel nutraceutical approach for the treatment of ACLD and portal hypertension based on published pre-clinical data.

## 1. The Liver: Function and Cell Components

The liver is a dynamic and complex organ that is crucial for the regulation and homeostasis of the organism. Both its size—as it is the largest organ of the human body—and its essential location make it a centerpiece for many physiological processes. The liver is the only organ with a dual blood supply: it receives oxygenated blood directly through the hepatic artery that derives from the celiac artery, and nutrient-rich blood coming from the drainage of other visceral organs, such as the intestines, through the portal vein [1]. Thus, it participates in many essential processes including macronutrient metabolism, blood volume regulation, endocrine control of growth pathways, lipid and cholesterol homeostasis and the processing of xenobiotic substances, among others [2].

### 1.1. The Hepatic Lobule

At the microscopic level, the liver is organized in hexagonal functional units called hepatic lobules. A total of 80% of their volume is made up of hepatocytes, which constitute the liver parenchyma. Hepatocytes carry out most hepatic metabolic and endocrine processes and are responsible for the secretion of many products [3]. At each vertex of a hepatic lobule a portal triad is located, which consists of the hepatic artery, the portal vein and biliary conducts, whereas at the center of the lobule is the central vein. Bile is released by hepatocytes and flows through bile canaliculi towards the edges of the hepatic lobule, where it is collected in the bile ducts. Blood, on the other hand, is provided by both the hepatic arteriole, which supplies highly oxygenated blood, and the portal venule, which supplies nutrient-rich blood. Blood from both origins is mixed together in a special capillary bed named hepatic sinusoid and flows towards the center of the lobule, where it is collected by the central vein [2,3]. Hepatocytes are lined up along the sinusoids, leaving the perisinusoidal space in between, also termed the space of Disse. Into this space, hepatocytes extend their microvilli that take up the oxygen and nutrients carried in the bloodstream while releasing metabolites and waste products. This leads to a phenomenon called zonation: a gradient of blood composition and, consequently, of the processes carried out by hepatic cells, between the outer and inner areas of the hepatic lobule. Even though these gradients are flexible and may be altered depending on nutritional state, in general, hepatocytes located in the outer zone will favor oxidative metabolism when compared to hepatocytes closer to the central vein, while the latter have a higher capacity to absorb free fatty acids [4,5].

### 1.2. Sinusoidal Cell Components

These complex and well-balanced processes are possible thanks to the particular characteristics of the hepatic sinusoids, which are highly permeable and specialized capillary vessels. They are composed of several non-parenchymal cell types such as liver sinusoidal endothelial cells (LSEC), hepatic stellate cells (HSC) and Kupffer cells (KC).

LSEC represent 15–20% of liver cells, but only 2.5% of its volume [6]. They form the endothelial monolayer around the sinusoids. Unlike other endothelial cells, LSEC present specific characteristics such as a lack of an organized basal membrane and the presence of transcytosolic pores of less than 200 nm in diameter called fenestrae. Those allow the circulation of oxygen, nutrients and other products between the bloodstream and the liver parenchyma, while maintaining a certain barrier function. Besides this key role, they also participate in other essential processes like inflammation, endocytosis and vascular tone regulation [6,7,8].

HSC can be found in the space of Disse or perisinusoidal space, and present cytosolic prolongations that confer them a star-shaped geometry. They represent 5–8% of hepatic cells [6]. In a healthy liver, HSC are in a quiescent state. They store vitamin A in cytosolic lipid droplets, control retinoid metabolism and regulate the growth and metabolism of neighboring cells, although they might also participate in other processes that are still not fully understood. In the context of sustained liver injury, HSC become activated, losing the vitamin A droplets and transdifferentiating into fibroblasts. Under this phenotype, HSC exhibit increased contractility and migration, uncontrolled proliferation and exacerbated extracellular matrix (ECM) deposition, being therefore responsible for hepatic fibrosis [9,10,11].

Lastly, KC are liver resident macrophages. Under physiological conditions, they represent around 6% of total liver cells [6]. Depending on the stimuli they detect, they can acquire a pro or anti-inflammatory phenotype. KC play a key role in inflammation, phagocytosis, antigen presentation and the secretion of soluble mediators [12].

## 2. Liver Disease: Relevance of ACLD and Portal Hypertension

Liver disease accounts for approximately 2 million deaths per year, constituting an important clinical problem worldwide. Of those, 1.03 million deaths are due to advanced chronic liver disease (ACLD) and its complications [13].

Chronic alcohol abuse [14], unhealthy dietary habits, such as those with a high content of fat or fructose [15,16], and hepatitis B and C virus infection [17] are the three leading causes of chronic liver disease (CLD). Even though the nature of these insults is different, when sustained over time they all lead to a state of perpetual tissue inflammation, parenchymal cell death and exacerbated deposition of ECM, ultimately causing hepatic fibrosis. Over the years, fibrosis can progress to cirrhosis, which is characterized by the development of aberrant regenerative nodules and fibrotic septa in the parenchyma, and even to hepatocellular carcinoma [18,19,20]. One of the most clinically relevant syndromes associated with ACLD is portal hypertension, which is considered clinically significant when the hepatic venous pressure gradient (HVPG)—an estimate of the pressure gradient between the portal vein and the inferior vena cava—increases above 10 mmHg. Portal hypertension is directly related to the development of dreadful complications such as the formation of portal-systemic collaterals, variceal bleeding, hepatic encephalopathy, ascites, jaundice, bacteremia, hypersplenism, and hepatorenal syndrome, among others, and represents the first non-neoplastic cause of death and the need for a liver transplantation in patients with ACLD [21].

From a clinical point of view, CLD progression can be classified into four stages: (1) compensated cirrhosis without varices, (2) compensated cirrhosis with varices, (3) decompensated cirrhosis with ascites, and (4) decompensated cirrhosis with variceal bleeding [20,22,23,24]. Very often, the first stages of the disease are asymptomatic, and CLD is only detected once there is a decompensated cirrhosis with severe portal hypertension (HVPG > 16 mmHg). This constitutes an additional challenge for therapy development, as such advanced stages of cirrhosis are more difficult to reverse and constitute a higher health risk. Indeed, despite extensive preclinical and clinical research in the last decades, current therapeutic options for the treatment of portal hypertension are limited, with a lack of a safe and effective enough treatment in clinical scenarios. Several therapeutic approaches have been—and are still being—investigated, both at the pre-clinical and clinical levels, including antifibrotic, vasoprotective and antioxidant strategies, among others [25,26,27]. However, liver transplantation remains a frequent treatment option for patients with ACLD [13].

Moreover, changes in nutrition and life habits over the last decades, have caused a rise of non-alcoholic fatty liver disease (NAFDL), with a worldwide prevalence of 25% in the general population. In patients suffering from obesity, diabetes or other metabolic risk factors, the prevalence of NAFLD is even higher, over 70% [28]. This is mainly caused by the increasing amounts of circulating free fatty acids and the chronic low-grade inflammation observed in the white adipose tissue [29]. Approximately one third of NAFLD patients progress to non-alcoholic steatohepatitis (NASH), and, as with other CLD etiologies, can ultimately develop cirrhosis and hepatocellular carcinoma [30].

Therefore, in spite of some encouraging advances, liver diseases leading to ACLD are increasingly challenging for public health. For this reason, further research efforts are needed to improve the current therapeutic landscape for ACLD.

## 3. Major Contributors of ACLD Pathophysiology

### 3.1. Inflammation

In physiological conditions, the liver can be exposed to pathogens carried in portal blood. Resident KC are able to detect their presence through their surface toll-like and nod-like receptors (TLR, NLR), and therefore constitute a first-line of defense for the liver.

**Figure 1:**
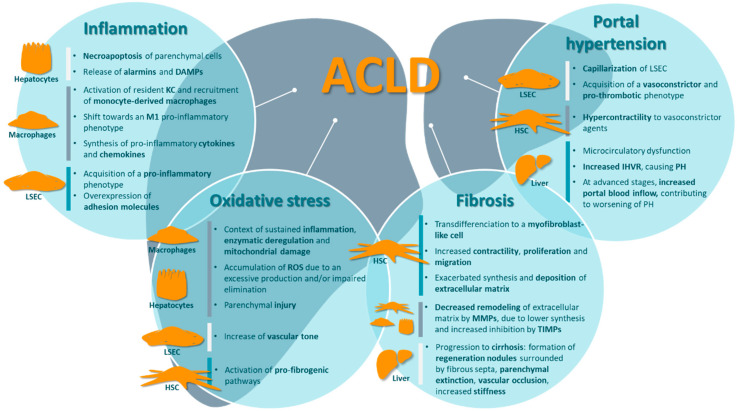
Major contributors of advanced chronic liver disease pathophysiology. ACLD involves a profound de-regulation of all hepatic cell types, including hepatocyte necroapoptosis, the shift of hepatic macrophages towards a pro-inflammatory phenotype, hepatic stellate cell activation, and liver sinusoidal endothelial cell capillarization. Altogether, these alterations contribute to a state of increased inflammation and oxidative stress, leading to the development of fibrosis and, ultimately, portal hypertension. ACLD, advanced chronic liver disease; DAMPs, damage-associated molecular patterns; HSC, hepatic stellate cells; IHVR, intrahepatic vascular resistance; KC, Kupffer cells; LSEC, liver sinusoidal endothelial cells; MMPs, metalloproteinases; PH, portal hypertension; ROS, reactive oxygen species; TIMPs, tissue inhibitors of metalloproteinases.

During CLD, damaged parenchymal cells undergo apoptosis or necroapoptosis releasing signals such as alarmins or damage-associated molecular patterns (DAMPs; Figure 1). KC are able to detect these signals and initiate an inflammatory response, notably through a TLR4-mediated mechanism that directly activates the canonical nuclear factor kappa-light-chain-enhancer of activated B cells (NFκB) signaling pathway [31,32]. Moreover, TLR activation in KC and HSC triggers monocyte recruitment to the liver through an increase in chemokine C-C motif ligand 2 (CCL2) and chemokine C-X-C motif ligand 1 (CXCL1) levels [33,34]. Those monocytes differentiate into macrophages and participate in hepatic inflammation. Consequentially, not only are liver macrophages heterogeneous in their origin, but they also exhibit distinct functions. While KC play a key role in initiating inflammation, monocyte-derived macrophages are linked to chronic inflammation and fibrogenesis [35].

In general, macrophages can acquire an M1 pro-inflammatory phenotype or an M2 anti-inflammatory phenotype. In response to pro-inflammatory stimuli, macrophages release cytokines, such as tumor necrosis factor alpha (TNF-α) or interleukins (IL) IL-1β, IL-6, IL-12, IL-18, and chemokines, that will, in turn, act as pro-inflammatory signals themselves. Said cytokines and chemokines can be captured by the neighboring inflammatory cells, magnifying the pro-inflammatory response, or by other cell types, giving rise to an intercellular cross-talk. M2-macrophages, on the other hand, secrete anti-inflammatory cytokines and growth factors, such as IL-10, transforming growth factor-β (TGF-β), platelet derived growth factor (PDGF) and epidermal growth factor (EGF), and participate in wound healing [12,36,37]. Nonetheless, the dichotomic view of M1 versus M2 phenotypes is nowadays considered a simplification of reality, as cells are usually exposed to a complex combination of pro- and anti-inflammatory signals, giving rise to a whole spectrum of activation states and functions [38].

Besides macrophages, other inflammatory agents participate in the inflammatory response, with neutrophils, dendritic cells and T-lymphocytes being the most relevant. Neutrophils are rapidly recruited to the liver after parenchymal injury and contribute to CLD progression by releasing both pro-inflammatory mediators and reactive oxygen and nitrogen species [39]. Furthermore, a recent study linked the presence of neutrophil extracellular traps (NETs) to microvascular thrombosis and portal hypertension in murine models of ACLD [40]. While dendritic cells’ and lymphocytes’ implications in the pathophysiology of ACLD are not yet fully understood, it is believed that they promote hepatocyte injury through the synthesis of inflammatory mediators or, in the case of T helpers 1, 2, 17 (Th1, Th2, Th17) even by directly killing hepatocytes [39,41].

Finally, it is important to note that inflammation can have beneficial or detrimental effects depending on the stage of the disease. Whereas in the early stages of CLD, chronic inflammation is mostly harmful, causing hepatocyte injury and fibrogenesis, and contributing to disease progression, in advanced stages of liver disease it can have positive effects by fighting frequent bacterial infections and stimulating liver regeneration [41].

### 3.2. Oxidative Stress

Reactive oxygen species (ROS) are highly chemically reactive molecules derived from oxygen metabolism. They can be found as free radicals, such as the superoxide anion (O_2_^−^) and hydroxyl radicals (OH^−^), or as non-radical forms, such as hydrogen peroxide (H_2_O_2_). ROS can be generated by incomplete reduction of oxygen during respiration or as by-products of metabolic processes carried out by enzymes like nitric oxidase synthase (NOS), nicotinamide adenine dinucleotide phosphate (NADPH) oxidase, cyclooxygenase (COX), or superoxide dismutase (SOD), among others [42]. Hepatocytes, HSC, LSEC, and KC are all capable of generating ROS. At low levels, ROS participate in many physiological processes, including intracellular messaging, regulation of cell growth, differentiation, and apoptosis. Even under non-pathological conditions, the liver is often exposed to toxic substances present in portal blood, causing an increase in ROS generation. For this reason, the antioxidant response in the liver is essential and highly regulated to ensure homeostasis [43].

In CLD, increased inflammation, enzymatic de-regulation and mitochondrial damage lead to an unbalance between ROS production and elimination through antioxidant mechanisms, causing their accumulation (Figure 1). This gives rise to a state of oxidative stress, in which ROS participate in the uncontrolled oxidation of lipids, proteins and DNA molecules, therefore causing severe cell damage. In this manner, ROS cause parenchymal injury, even inducing hepatocyte death, and further contribute to inflammation and CLD progression. ROS can also lead to the accumulation of reactive nitrogen species (RNS), such as peroxynitrite anions (ONOO^−^), which can be equally damaging for the cells and participate in many pathogenic processes [44,45,46]. Moreover, the presence of ROS can trigger signaling cascades that affect transcriptional regulation, notably through nuclear factor erythroid 2-related factor 2 (Nrf2). Some of these responses constitute homeostatic mechanisms against oxidative stress, by inducing the expression of antioxidant agents (i.e., catalase, glutathione S-transferase, glutathione peroxidase or heme oxygenase-1), but these fail to compensate for excessive ROS accumulation under pathological conditions [47].

Furthermore, ROS can specifically activate signaling pathways in HSC and LSEC, promoting fibrogenesis and increasing vascular tone. Particularly, in HSC, it has been suggested that ROS can trigger c-Jun N-terminal kinase (JNK), NFκB, and TGF-β signaling pathways, increasing the expression of collagen and other ECM components as well as pro-inflammatory mediators [48,49,50,51]. However, LSEC are probably the most sensitive liver cell type to oxidative stress. In response to ROS accumulation, they overexpress adhesion molecules, such as selectins, vascular cell adhesion molecule-1 (VCAM-1), and intercellular adhesion molecule-1 (ICAM-1), secrete pro-inflammatory mediators, and can even undergo autophagy as a mechanism to maintain homeostasis [6]. Moreover, in LSEC, the presence of O_2_^−^ contributes to the depletion of nitric oxide (NO) bioavailability, both through an increased scavenging and a reduced synthesis of NO. On one hand, O_2_^−^ reacts with NO producing ONOO^−^ while, at the same time, it inhibits endothelial NOS (eNOS) activation by reducing its phosphorylation and increasing its inhibitors. This phenomenon favors the capillarization of LSEC and endothelial dysfunction [44,52,53].

### 3.3. Fibrosis

Hepatic fibrosis is defined as the excessive accumulation of ECM proteins, such as collagen, fibronectin or elastin, in the liver. It is a highly conserved response to tissue damage and exerts beneficial effects in acute injury by preventing its propagation. However, when injury is sustained over time, the fibrogenic response can become detrimental to the point of completely disrupting liver parenchymal and vascular architecture [54].

During CLD, in response to liver injury, inflammation, and oxidative stress, HSC become activated and transdifferentiate from a quiescent state to an α-smooth muscle actin (α-SMA)-positive myofibroblast-like cell (Figure 1). In their activated form, HSC exhibit increased proliferation, contractility, migration and, most importantly, an exacerbated synthesis and deposition of ECM components, constituting the main driver of hepatic fibrosis. There is a wide variety of signals and pathways implicated in this phenomenon. However, two of the most central mechanisms of HSC activation are the canonical TGF-β and PDGF signaling pathways [55]. In addition to an increased ECM synthesis, fibrotic livers also present a decreased remodeling by metalloproteinases (MMPs), further contributing to ECM accumulation. This is due both to a reduction of MMPs expression, and to a decreased activity mainly through an overexpression of their specific inhibitors (TIMPs) [56].

With time, fibrosis can progress to cirrhosis, with the development of characteristic fibrous septa surrounding areas of preserved parenchyma termed regeneration nodules. This level of architectural distortion seriously hinders blood circulation through the liver and can entail major complications such as portal hypertension [57]. Even though fibrosis was traditionally considered irreversible, it has since been shown that, at early stages, complete resolution can be achieved. Even at advanced stages of cirrhosis, a certain degree of regression is possible, markedly improving prognosis and, thus, raising interest for anti-fibrotic therapies [27,58,59,60,61].

### 3.4. Portal Hypertension and Other Complications

ACLD is characterized by a profound de-regulation of all hepatic cell types. As mentioned previously, hepatocytes undergo necroapoptosis, hepatic macrophages are polarized towards a pro-inflammatory phenotype, LSEC experience capillarization, and HSC become activated and acquire a pro-fibrogenic phenotype. Taken together, these alterations are responsible for the two main factors leading to portal hypertension. On one hand is the structural component: fibrosis, vascular occlusion, and the development of regeneration nodules contributing to the mechanical obstruction of blood flow through the liver. On the other hand is the dynamic component: an unbalance between vasoconstrictor and vasodilator synthesis, together with a higher sensitivity to vasoconstrictors, leading to sinusoidal hypercontractility (Figure 1) [57,62].

According to Ohm’s law, ΔP = Q · R, the pressure gradient between two points in a fluidic system (ΔP or, in our case, HVPG) is given by the flow (Q) and the resistance of the system (R). During CLD’s progression, the primary factor involved in the development of portal hypertension is an increase in intrahepatic vascular resistance (IHVR), or R. Approximately, the structural and dynamic components are responsible for 60% and 40% of said increase, respectively. As opposed to anti-fibrotic therapies, which require a certain amount of time to exert noticeable effects, the dynamic component offers the opportunity to acutely modulate portal pressure [63], constituting an attractive therapeutic target. However, at more advanced stages of the disease, an increase in portal blood flow, or Q, takes place due to mesenteric arterial vasodilation, contributing to the worsening of portal hypertension [64,65].

Lastly, portal hypertension entails a considerable number of severe health complications. Approximately 50% of ACLD patients develop varices due to the diversion of up to 90% of portal flow through portal-systemic collaterals. These enlarged and thin-walled vessels are extremely fragile and can easily rupture, causing variceal hemorrhage [66,67]. Some complications of portal hypertension arise from an increased gut permeability, which can lead to hepatic encephalopathy or other systemic infections. Finally, other frequent complications include ascites, hepatorenal syndrome, splenomegaly and hepatopulmonary syndrome.

## 4. TG-DHA as a Novel Nutraceutical Approach

### 4.1. TG-DHA’s Mechanism of Action and Demonstrated Benefits for the Human Health

ω-3 fatty acids are a family of essential polyunsaturated fatty acids (PUFAs) characterized by the presence of a double bond three atoms away from their terminal methyl group. α-Linolenic acid (ALA) is the precursor of this family and can be converted, through a series of desaturation and elongation reactions, to the more biologically active long-chain ω-3 eicosapentaenoic acid (EPA) and docosahexaenoic acid (DHA). Although intake of ALA could theoretically be sufficient for EPA and DHA synthesis, as the human body is able to carry out the subsequent reactions, ALA conversion to ω-3 PUFAs has been reported to be actually very limited [68]. For this reason, ω-3 PUFAs must be obtained from diet. Some of the most important dietary sources of ω-3 are species of fatty fish, like mackerel, herring, salmon, tuna, and sardines, or the liver of some species of lean fish, such as cod [69].

DHA is the end-product of the ω-3 synthesis pathway and, therefore, the most difficult to obtain [70,71]. TG-DHA (triglycerides of DHA) in particular, present a higher intestinal absorption and bioactivity when compared to similar dietary supplements, thanks to their triglyceride form and the preservation of the cis structure of their double bonds. These characteristics confer TG-DHA a great potential as a nutraceutical, both in healthy and diseased states. Indeed, DHA is highly pleiotropic and participates in a wide range of physiological roles, from maintaining cardiovascular health to constituting a structural component of the brain and central nervous system [72]. In particular, DHA possesses strong anti-inflammatory and anti-oxidant effects. On one hand, it directly regulates key transcription factors such as peroxisome proliferator-activated receptor gamma (PPAR) and NFκB, inhibiting the expression of pro-inflammatory cytokines TNF-α, IL-1, IL-6, IL-8, and IL-12, inducible NOS, and COX-2, as well as regulating metabolism. It can also bind to some G-protein coupled surface receptors (GPR), such as GPR120, which is involved in anti-inflammatory signaling. Moreover, DHA has been implicated in inflammation resolution by pro-resolving lipid mediators such as resolvins and protectins, inhibition of eicosanoid synthesis, reduction of O_2_^−^ levels, decreased lipid peroxidation, and activation of antioxidant pathways [72,73,74,75]. TG-DHA, in particular, exhibits a high antioxidant activity and has been patented for its use in cellular oxidative damage prevention [76]. 

Since inflammation and oxidative stress play a key role in many diseases’ pathophysiology’s, TG-DHA constitutes a promising dietary supplement that could be used in very diverse medical fields. In fact, TG-DHA has shown to exert beneficial effects in neurodegenerative diseases, exhibiting a neuroprotective effect in experimental parkinsonism [77] and improving autoimmune encephalomyelitis in mice [78]. Moreover, it has been reported to ameliorate macular function in diabetic retinopathy [79], behavioral parameters in patients with attention-deficit hyperactivity disorder (ADHD) [80], and even sperm quality by decreasing its DNA fragmentation [81].

### 4.2. TG-DHA in ACLD 

**Figure 2:**
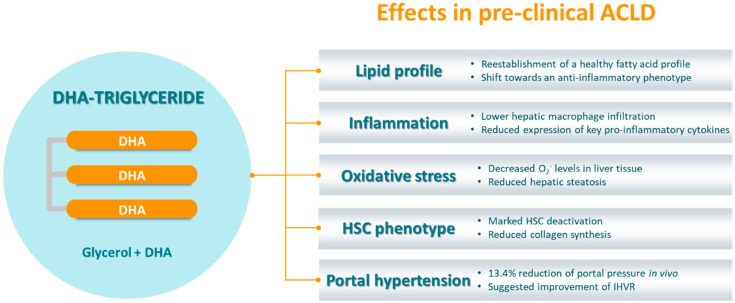
DHA triglyceride structure and demonstrated effects in an animal model of advanced chronic liver disease. Treatment with DHA triglycerides (TG-DHA) improved many aspects of ACLD pathophysiology in a rat model of the disease, including hepatic fatty acid profile, inflammation, oxidative stress, HSC phenotype and portal hypertension. ACLD, advanced chronic liver disease; DHA, docosahexaenoic acid; HSC, hepatic stellate cells; IHVR, intrahepatic vascular resistance; O_2_^−^, superoxide anion.

In a previous publication from our research group [82], we found that ACLD’s pathophysiology involves a profound deregulation of the hepatic fatty acid profile. The analysis, performed in livers from healthy and cirrhotic rats, showed that, among other alterations, cirrhotic livers are markedly depleted in DHA. In fact, they present an unbalance between ω-3 and ω-6 PUFAs that corresponds to a pro-inflammatory phenotype, with increased arachidonic acid (AA)/DHA and AA/EPA ratios. Interestingly, treating cirrhotic rats with TG-DHA reestablished a healthy fatty acid profile, including a recovery of membrane characteristics and a shift to an anti-inflammatory phenotype (Figure 2).

Based on TG-DHA’s beneficial effects on hepatic fatty acid profile, and given its anti-inflammatory and anti-oxidant properties, we hypothesized that it could equally improve other aspects of ACLD. Indeed, cirrhotic rats that received TG-DHA exhibited a significant improvement of portal hypertension (−13.4% in portal pressure in vivo) without changes in portal blood flow, thus suggesting an amelioration of IHVR. Reduction of portal pressure in such advanced stages of CLD is linked to a significant improvement in prognosis [83], and is an indicator of underlying phenotypic ameliorations.

Subsequent analyses revealed that cirrhotic rats treated with TG-DHA presented reduced steatosis and oxidative stress (measured as O_2_^−^ levels in liver tissue). In agreement with these results, a reduction in hepatic steatosis has been previously linked to long-chain ω-3-driven oxidative stress inhibition in an experimental model of high-fat diet [84]. TG-DHA-treated rats also exhibited reduced inflammation, with lower hepatic macrophage infiltration and expression of key pro-inflammatory cytokines. Furthermore, complementary in vitro studies performed with murine macrophages evidenced that TG-DHA grants a protective effect against future inflammatory challenges, suggesting a possible beneficial outcome of preventive or early-stage dietary supplementation.

HSC phenotype analysis revealed a significant deactivation, as well as a reduction of collagen synthesis. However, TG-DHA failed to statistically significantly improve liver fibrosis, probably due to insufficient ECM remodeling, underlining the necessity to extend the treatment period (of merely 2 weeks in this study) or to combine TG-DHA with complementary anti-fibrotic therapies. Moreover, an unchanged structural component would suggest that portal hypertension improvement is related to an amelioration of the dynamic component of ACLD, which is closely associated to oxidative stress and inflammation. In fact, ω-3 PUFAs have been reported to ameliorate hepatic microcirculation in a rat model of ischemia/reperfusion [85].

Lastly, HSC deactivation was confirmed in vitro in human LX2 cells, suggesting TG-DHA’s translatability and encouraging its evaluation at the clinical scenario.

## 5. Future Perspectives

The current dietary trend, especially in western countries, favors the consumption of ω-6 over ω-3 PUFAs, causing an unbalance between these two families of essential fatty acids that ultimately leads to a state of increased oxidative stress and inflammation [72,86]. Given that ω-3 PUFAs are essential constituents of all human cells and possess an excellent safety profile, TG-DHA constitutes an attractive nutraceutical product that can be used both in healthy and pathological contexts. 

As in many other diseases, oxidative stress plays a key role in the pathogenesis of ACLD, contributing to the deregulation of all hepatic cell types and the progression of fibrosis. Indeed, the use of TG-DHA for the treatment of experimental ACLD has shown promising results [82]. However, given the complexity of the disease, the therapeutic approach for ACLD has recently shifted from monotherapy to combination therapy. While TG-DHA has already been combined with caffeine as a strategy for the treatment of Parkinson’s disease [87], evaluating its combination with vasomodulator or anti-fibrotic drugs would be of great interest for the treatment of ACLD and portal hypertension.
